# Tubarial salivary glands on PSMA ligands based PET imaging and post ^177^Lu PSMA therapy scan: reiterating its importance

**DOI:** 10.22038/AOJNMB.2023.72138.1505

**Published:** 2024

**Authors:** Srinivas Ananth Kumar, Anjali Meena, Ashwani Sood, Rajender Kumar, Bhagwant Rai Mittal

**Affiliations:** Department of Nuclear Medicine, Post Graduate Institute of Medical Education and Research, Chandigarh, India

**Keywords:** Tubarial salivary glands, PSMA, PET/CT, Lu-177 PSMA-617

## Abstract

^68^Ga-PSMA PET/CT has been routinely utilized in patients with intermediate to high-risk category prostate carcinoma for staging, biochemical recurrence and before planning the PSMA radioligand therapy (RLT). ^177^Lu-PSMA RLT has also been approved by FDA as a novel treatment modality in metastatic carcinoma prostate patients who have failed to other lines of treatment. The non-target organs like salivary and lacrimal glands have shown to have high physiological PSMA uptake on PSMA PET/CT. Recently, strong uptake of PSMA ligand has also been noted in the dorsal wall of the nasopharynx in the region of torus tubarius on PSMA PET/CT, which has led to the identification of new pair of salivary gland structures called “tubarial salivary glands”. The clinical significance of these distinct anatomical structures lies in the fact these structures might be involved in a variety of immune related, inflammatory disorders, malignancies and could be a probable organ at risk during radiotherapy in case of head and neck malignancies, causing adverse effects to the patient.

## Introduction

^68^Ga-PSMA PET/CT guided ^177^Lu PSMA RLT has revolutionized the treatment of metastatic carcinoma patients refractory to other lines of management with the results of VISION trial (1). 

 PSMA ligand uptake has been recently demonstrated in posterior nasopharynx in a distinct pair of anatomical structures called “tubarial salivary glands” in addition to the physiological tracer in the major salivary and lacrimal glands. Though the uptake in the tubarial glands might be lower compared with other major salivary glands, yet it is comparable with that of the tumor which could result in false interpretation of PSMA PET/CT. Here we present incidental tracer uptake in the tubarial salivary glands in a metastatic carcinoma prostate patient on ^68^Ga-PSMA PET/CT and in the subsequent ^177^Lu PSMA-617 post-therapy images.

## Case Report

 A 68-year-old male presented with complaints of lower urinary tract symptoms with clinical suspicion of benign prostatic hyperplasia, underwent transurethral resection of prostate. 

 The histopathology specimen showed adenocarcinoma prostate, Gleason score 4+3. 

**Figure 1:**
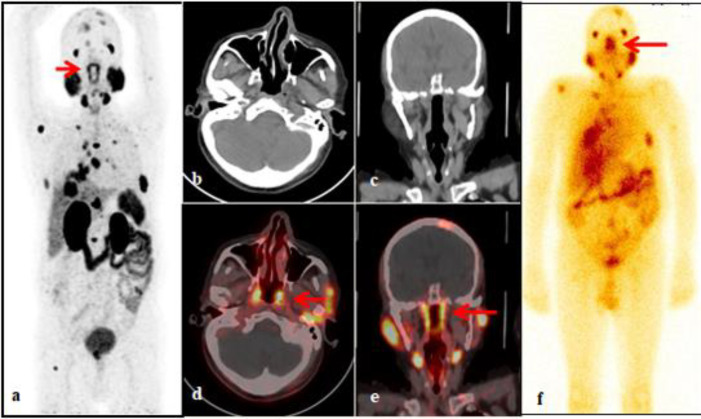
(**A-F**): ^68^Ga-PSMA PET/CT in a proven carcinoma prostate patient-maximum intensity projection image showing tracer avid lesions in the prostate, multiple skeletal lesions (skull, sternum, scapula, rib, multiple cervicodorsolumbar vertebrae, left iliac bone and right femur), mediastinal lymph nodes, fibro-consolidatory lesion in the lower lobe of the right lung and additional tracer uptake in nasal region (**arrow**) (**a**). Axial & coronal CT and corresponding fused PET/CT images of head showing physiological tracer uptake in the major and minor salivary glands, lacrimal glands with additional intense tracer uptake in the region of posterior pharynx likely in the tubarial salivary glands (**arrows**) (**b-e**). Anterior post-therapy whole-body image (post ^177^Lu PSMA-617 administration) showing tracer uptake in the tubarial salivary glands (**arrow**) and at other sites similar to the tracer distribution on ^68^Ga-PSMA PET/CT (**f**)

 Subsequently patient underwent bilateral orchidectomy. On follow-up, patient was started on bicalutamide followed by abiraterone- prednisone therapy. In view of disease progression, patient was referred to our department for ^68^Ga-PSMA PET/CT for staging and planning for ^177^Lu-PSMA radionuclide therapy. ^68^Ga-PSMA PET/CT showed tracer uptake in the prostate, and multiple other metastatic sites. In addition to that, intense tracer uptake was noted in the region of posterior pharynx along with physiological tracer uptake in the major & minor salivary glands and lacrimal glands (Figure 1 a-e). After assessing the patient’s eligibility for radionuclide therapy, 200 mCi of ^177^Lu PSMA-617 was administered to the patient under supervision after adequate hydration. Post therapy images demonstrated tracer uptake in the region of posterior nasopharynx and at other sites similar to the distribution on ^68^Ga-PSMA PET/CT (Figure 1 f). The tracer uptake in the region of nasopharynx posteriorly on both ^68^Ga-PSMA PET/CT and ^177^Lu PSMA – 617 post therapy images corresponded to clinically relevant new pair of anatomical structures called “tubarial salivary glands”.

## Discussion

 PSMA ligands show intense physiological tracer uptake within the major and minor salivary glands (2, 3). Valstar et al (4) in their study brought to light a new pair of clinically relevant salivary glands in the region of the torus tubarius in the posterior wall of the nasopharynx by using ^68^Ga-PSMA PET/CT, for which the terminology “tubarial glands” was propounded. The description of these glands did not fit to any anatomical structures previously known to the scientific community. 

 Cadaveric studies with histology coupled with the subsequent PSMA imaging studies have exemplified that the tubarial salivary glands are bilateral macroscopic glands in the posterior nasopharynx draping over the torus tubarius helping in the lubrication of the nasopharynx and oropharynx. The clinical significance of these tubarial salivary glands lies in the fact that they might be involved in a variety of clinical disorders like IgG4 related disease, Sjogren syndrome and even malignancies (5,6). 

 Detection of these salivary glands have been difficult with conventional imaging modalities like CT, MRI and also with ^99m^Tc pertechnetate salivary scintigraphy (7). However, PSMA based ligands PET/CT imaging have outperformed the imaging modalities like CT and bone scintigraphy in prostate cancer imaging with successful identification of these pair of salivary glands (8). Physiological uptake in these glands is usually less than the uptake in the major salivary glands but comparable to that of tumour metastatic disease. Hence physiological uptake within these glands must be borne in mind at staging, as well as to avoid false positive interpretation during therapy planning and at follow-up scans in patients with metastatic prostate carcinoma (9). Similarly, uptake within the tubarial salivary glands might also be seen on ^177^Lu-PSMA post-therapy scans and should not be falsely interpreted and counted with the overall disease burden. The patient in the current research work did not experience any adverse clinical effects, post-PSMA radionuclide therapy. The question arises and remains whether these newly discovered structures are of any interest to salivary gland toxicity related to the radionuclide therapy, with uptake in these new glands being concordant with the major salivary glands, requiring further studies. 

 These glands have been traditionally brushed aside during planning radiation therapy for head and neck malignancies with no due consideration as an organ at risk, till date. 

 Mapping these tubarial glands permits safer options for focused and targeted radiation treatment, aiming to preserve the function of these glands, improving the quality of life of these patients and avoiding/ minimizing complications like xerostomia and dysphagia. In addition, pathologists should also be aware of the existence of these tubarial salivary glands when interpreting pathologic lesions in the nasopharynx for precise diagnosis.

## Financial support for the work

 There is no financial disclosure or conflict of interest.
